# Prevalence of school bullying and its relationship with attention deficit-hyperactivity disorder and conduct disorder: a cross-sectional study

**DOI:** 10.1186/s41983-022-00494-6

**Published:** 2022-05-23

**Authors:** Gellan K. Ahmed, Nabil A. Metwaly, Khaled Elbeh, Marwa Salah Galal, Islam Shaaban

**Affiliations:** 1 Department of Neurology and Psychiatry, Faculty of Medicine, Assiut University, Asyût, Egypt; 2 Department of Child & Adolescent Psychiatry, King’s College London, London, SE5 8AF UK; 3 Department of Neurology, Al-Azhar University Hospital, Asyût, Egypt; 4 Department of Psychiatry, Sohag Psychiatric Hospital, Sohâg, Egypt

**Keywords:** School bullying, ADHD, Conduct, Risk factors

## Abstract

**Background:**

School bullying is the most widespread form of violence among adolescents. It has been identified as a critical problem for students and has evolved into a public health issue and global crisis. The study aims to assess the prevalence of school bullying among primary school students and its relationship with attention deficit hyperactive disorder (ADHD) and conduct disorders. Among the 280 primary school students those aged 10–12 years were recruited. All participants were assessed by parent interview, the Arabic version of the bullying behavior scale for children and adolescents and the Arabic version of the Conners’ Teacher Rating Scale-28.

**Results:**

We found that the prevalence rate of bullying behavior was 12.5% among students. In bullying students’ group, males were higher percentage (15.8%) than females (9%). Also, they had the highest mean scores regarding verbal bullying and social bullying, followed by psychological and physical bullying. Regarding Conners’, the higher mean scores of conduct problem, passive-inattentive, and hyperactivity index were associated with bullying students in compared to students without bullying.

**Conclusions:**

The prevalence of school bullying among primary school students was 12.5%. Also, there was significant association between bullying students and having attention deficit hyperactive disorder (ADHD) and/or conduct disorder.

## Introduction

Bullying, a common form of violence among teenagers, is a serious issue for students as well as the wider public health community [1]. Bullying is defined as repeated and chronic negative actions directed at a student or group of students, characterized by a power imbalance between the aggressor and the victim [2, 3]. School bullying is described as a sort of violence that harms others and occurs when a student or a group of students use their strength to harm other individuals or other groups while at school or participating in various activities [4]. The most common types of bullying are physical (hitting, shoving, stealing, or damaging property), verbal (name-calling, mocking, or hurtful teasing), social (excluding others from a group, spreading rumors, or damaging friendships), sexual (dirty words, touch, or making sexist comments), and electronic or cyber-bullying (spreading rumors and hurtful comments via cell phones [e.g., text messaging], email, or social media) [5].

According to the Global School-Based Student Health Survey, an international survey conducted among middle school students between 2006 and 2008, 60% of students in Egypt and 33% of students in Libya, Morocco, and Tunisia reported being bullied in the previous month [6]. Among the 9- to 12-year-old age group, 49.8% reported being bullied at school, and 14.5% reported being bullied online [7].

Several risk factors may encourage school bullying. These risk factors are categorized into individual, peer, school, parental, community, and societal risk factors [8]. Regarding the individual risk factors, underweight and obese children [9], belonging to higher socioeconomic status [10], being male [11], poor academic performance [12] are more common characteristics to be victim with bully behavior. In addition, peer risk factors may include not conforming to peer-group norms [13] and having a delinquent record [14] are associated with bullies. School risk factors could be determined the bully behavior regarding the school environment [15] and teachers’ responses to bullying behavior [16]. Children with bully behavior may also have experienced physical abuse and being from low socioeconomic status families with authoritarian parents [17].

Some researchers believe that psychopathology and bullying go hand in hand, while others disagree. There is evidence pointing to a bidirectional relationship between psychopathology and bullying. According to Hwang and Kim [18], children who exhibit disruptive behavior, such as attention-deficit/hyperactivity disorder (ADHD), may be more likely to engage in peer violence. A study conducted in Finland found that ADHD was the most common psychiatric disorder among children who were bullied [19]. Several studies investigating risk factors for bullying also reported that children with ADHD are more likely than neurotypical children to be involved in bullying, either as aggressors or victims [20–22]. Bullying is also a predictor of involvement in a wide range of violent and nonviolent behaviors. Individuals with a history of bullying were nearly 11 times more likely than nonbullying peers to be diagnosed with conduct disorder and nearly eight times more likely to meet the criteria for antisocial personality disorder, according to a multivariate analysis that controlled for sociodemographic, lifetime psychiatric disorders, and family history of antisocial behavior [23].

Students who are bullied at school are more likely to experience serious psychological, social, academic, and mental health problems [24]. Thus, more research into bullying is needed to facilitate the development of effective intervention programs. To prevent violence and bullying, we must first have a clear understanding of them. To better understand bullying, we examined the qualities and characteristics of people involved in bullying. We assessed the prevalence rate and possible risk factors for school bullying among primary school students. Additionally, we investigated the relationships between school bullying and ADHD and conduct disorder.

## Methods

### Participants

The present study included 280 randomly selected primary school students (146 males and 134 females) recruited from Ali bin Abi Talib Primary Azhar School, Sohag Governorate, Egypt. Data were collected between November 2019 to the end of March 2020. We included children aged 10–12 years (4th, 5th, and 6th primary grades) who completed the questionnaire. We excluded children who had histories of neurological or medical diseases or previous psychiatric disorders.

Data were collected in two phases. In stage 1, we screened for bullying behavior, and in stage 2, we studied the risk factors for bullying and its comorbidity with ADHD and conduct disorder. The sample size was estimated using the EPI info statistical package Version 7. The used parameters for determining sample size were a proportion of 0.5, a confidence level of 95% and a margin of error of 5%. So, we recruited 280 students in stage 1. All participants were screened for bullying behavior using the Arabic version of the Bullying Behavior Scale for children and adolescents [25]. For stage 2, 35 students who screened positive for bullying were selected as the study group, and another 35 students were randomly selected as the control group. The teacher had asked these students’ parents to come to school to be interviewed and assessed for possible risk factors of bullying, ADHD, and conduct disorder using parent interviews by The Kiddie Schedule for Affective Disorders and Schizophrenia-Present Version (K-SADS-P) and appropriate scales with teachers.

### Tools

1. Demographic data
  We collected data on age, gender, school grade, history of previous medical or psychiatric illness.
2. The Bullying Behavior Scale for children and adolescents [25]
  A self-administered questionnaire was used to screen for bullying behavior. The scale includes 40 statements that assess bullying subtypes: psychological bullying, verbal bullying, social bullying, and physical bullying. The lowest and highest possible total scores are 40 and 200, respectively [25]. We considered values higher than 80 to indicate significant bullying behavior.
3. Risk factor assessments
  We constructed a questionnaire to assess bullying risk factors following an extensive review of the available literature and related studies. The questionnaire consisted of 10 statements and was completed by the participants’ teachers. The 10 statements enquired about individual risk factors (not being accepted by peers, attention-seeking, tendency to be arrogant, school achievement, body build), peer risk factors (joining bad peer group, being bullied, tendency to imitate bad behaviors of peers), and school risk factors (being frequently punished by teachers, negative attitude toward teachers). All questions were answered by (yes/no) except school achievement and body-build questions. School achievement was evaluated based on midterm scores achieved by students (scores < 60% = weak, 60–70% = average, > 70% = good). Body build was evaluated according to BMI (< 18.5 = underweight, 18.5–24.9 = normal weight, > 25 = overweight).
4. The Kiddie Schedule for Affective Disorders and Schizophrenia-Present Version
  (K-SADS-P): A semi-structured diagnostic interviewing which both the kid and the parent are interviewed by experienced clinicians or clinical researchers. It evaluates symptoms that happened during the most recent episode (during the week before to the interview) as well as symptoms that occurred within the previous year [26].
5. Arabic version of Conners’ Teacher Rating Scale-28 [27]
  The 28-item behavior rating scales were completed by the participants’ teachers. The scale screening for behavioral markers associated with ADHD and conducts disorder. Responses are scored as follows: zero for “not at all,” 1 for “just a little,” 2 for “pretty much,” and 3 for “very much.” It translates Arabic by Abdul-Raqib Al-Behairy and Mustafa Abdul-Mohsen Al-Hudaibi [28].

### Statistical analysis

Data were revised, coded, processed, and analyzed using SPSS (Statistical Package for Social Science) for Windows version 25 (SPSS Inc., Chicago, IL, USA). Qualitative data were presented as numbers and percentages, and quantitative data were presented as means and standard deviations. The Chi-square test was used to compare qualitative between the two groups; Fisher’s exact test was used when the expected count in any cell was less than five. The two groups were compared using independent *t*-tests for quantitative data with parametric distribution, and the Mann–Whitney test for nonparametric quantitative data. The confidence interval was set to 95% and the accepted margin of error was set to 5% (*P* < 0.05 was considered significant).

## Results

### Sociodemographic data

A total of 280 students were recruited. The proportion of male students was slightly higher than that of female students at 52.1% and 47.9%, respectively. The mean age of participants was 11.08 ± 0.8 years. The majority of participants were in grade 6, followed by grade 5 and grade 4 (37.1%, 33.2%, and 29.6%, respectively) (see Table 1).

**Table 1:** Demographic data of the total participants

Item	Frequency	%
Sex		
Males	146	52.1%
Females	134	47.9%
Age		
Mean ± SD	11.08 ± 0.8	
Median (interquartile range)	11 (10–12)	
School grade		
Grade four	83	29.6%
Grade five	93	33.2%
Grade six	104	37.1%

### Prevalence of bullying behavior

Bullying behavior was exhibited by 12.5% of participants. There were significantly more male bullies than female bullies involved in all bullying types (Table 2). Males also had significantly higher mean scores in all bullying types, except social bullying.

**Table 2:** Distribution of types of bullying among bullied students

Bullying type	Mean ± SD Median (range)			*T* value	*p-value
	Total participation (*N* = 35)	Males (*N* = 23)	Females (*N* = 12)		
Verbal	2.5 ± 0.5	2.7 ± 0.4	2.1 ± 0.3	4.05	0.000
Psychological	2.5 ± 0.4	2.6 ± 0.3	2.3 ± 0.3	2.4	0.019
Social	2.5 ± 0.6	2.3 ± 0.6	2.9 ± 0.4	3.14	0.004
Physical	2.3 ± 0.7	2.6 ± 0.7	1.6 ± 0.4	4.47	0.000

^*^P value calculated by Mann–Whitney test

### Risk factors for school bullying

We assessed risk factors among 35 students who screened positive for bullying and another 35 randomly selected participants (nonbullies). There were statistically significant between the two groups (bully and nonbully) for all peer risk factors (except being bullied), all school risk factors, and one individual factors (not accepted by peers) (Table 3). The most significant risk factors for bullying in both groups were attention-seeking (48.6%) and imitating bad behaviors of others (48.6%), followed by being bullied (45.7%) and not accepted by peers (45.7%), negative attitude toward teachers (44.3%), and joining bad peer groups (42.9%). The bully group scored significantly higher in all risk factors than the nonbully group, except high scholastic achievement, underweight body build, and being bullied (see Table 3).

**Table 3:** Risk factors of bulling among studied groups

Variables	Bully group (*N* = 35) *N*%			Non-bully group (*N* = 35) *N*%			*P* value for males	Chi-square for males	*P* value for females	Chi-square for females	*P* value for both groups	Chi-square for both groups
	Males	Females	Total	Males	Females	Total						
Individual risk factors												
Not accepted from colleagues	17 (73.9%)	8 (66.7%)	25 (71.4%)	4 (17.4%)	3 (25%)	7 (20%)	0.000*	14.8	0.05	4.19	0.00*	18.65
Attention seeking	14 (60.9%)	7 (58.3%)	21 (60%)	7 (30.4%)	6 (50%)	13 (37.1%)	0.075	4.29	0.5	0.16	0.09	3.66
Tends to be arrogant	6 (26.1%)	5 (41.7%)	11 (31.4%)	3 (13%)	4 (33.3%)	7 (20%)	0.23	1.24	0.5	0.17	0.2	1.19
School achievement												
Low	9 (39.1%)	4 (33.3%)	13 (37.1%)	11 (47.8%)	2 (16.7%)	13 (37.1%)	0.65	0.84	0.41	1.7	0.47	1.5
Average	10 (43.5%	5 (41.7%)	15 (42.9%)	7 (30%)	4 (33.3%)	11 (31.4%)						
High	4 (17.4%)	3 (25%)	7 (20%)	5 (21.7%)	6 (50%)	11 (31.4%)						
Body built (BMI)												
Low	1 (4.3%)	2 (16.7%)	3 (8.6%)	7 (30.4%)	1 (8.3%)	8 (22.9%)	0.019*	7.9	0.76	0.53	0.114	4.3
Average	8 (34.8%)	8 (66.7%)	16 (45.7%)	10 (43.5%)	8 (66.7%)	18 (51.4%)						
High	14 (60.9%)	2 (16.7%)	16 (45.7%)	6 (26.1%)	3 (25%)	9 (25.7%)						
Peer risk factors												
Join bad peers’ groups	21 (91.3%)	2 (16.7%)	23 (65.7%)	6 (26.1%)	1 (8.3%)	7 (20%)	0.000*	20.17	0.5	0.38	0.00*	14.9
Being bullied	7 (30.4%)	8 (66.7%)	15 (42.9%)	12 (52.2%)	5 (41.7%)	17 (48.6%)	0.23	2.24	0.41	1.5	0.4	0.23
Imitation bad behavior of others	22 (95.7%)	6 (50%)	28 (80%)	5 (21.7%)	1 (8.3%)	6 (17.1%)	0.000*	25.91	0.069	5.04	0.00*	27.6
School risk factors												
Frequently punished by teacher	12 (52.2%)	1 (8.3%)	22 (62.9%)	0 (0%)	0 (0%)	0 (0%)	0.000*	16.23	0.5	1.04	0.00*	15.9
Negative attitude by teacher	18 (78.3%)	7 (58.3%)	25 (71.4%)	5 (21.7%)	1 (8.3%)	6 (17.1%)	0.000*	14.69	0.014*	6.75	0.00*	20.9

*significant *p* value

There were statistically significant between males in the two groups for two individual risk factors (not being accepted by peers and body build). All peer and school risk factors were statistically significant between males in the two groups, except being bullied. Among males, bullies had significantly higher percentages in all risk factors, except school achievement, body build, and being bullied compared to nonbully males. A higher proportion of male bullies had average school achievement and high body build, whereas nonbully males had higher proportions of low and high school achievement and low and average body build.

Among females, there were statistically significant in one school risk factor (negative attitude toward teacher) between the bully and nonbully groups. Among female students, bullies scored significantly higher on all subscales except high body build (BMI) and high school achievement.

Among bullies of both genders, the proportion of females was significantly lower on all subscales, except tends to be arrogant, being bullied, school achievement, and body build, compared to males. A higher percentage of bully females (41.7%) than bully males (26.1%) tended to be arrogant. High scholastic achievement was observed significantly more among bully females (25%) than males (17.4%). Low and average body build were significantly more common among bully females compared to bully males, whereas high body build was less common among females (16.7%) than males. In terms of being bullied themselves, more female bullies (66.7%) than male bullies (30.4%) experienced bullying from others.

Among nonbullies of both genders, a higher proportion of females was observed for all risk factors, except low school achievement, low and high body build, all peer risk factors, and negative attitude toward teachers.

### Relationship between bullying and ADHD and conduct disorder

Among males, there were significant differences between the bully and nonbully groups for all subscales of Conners’ Teacher Rating Scale-28 (Table 4); bullies scored significantly higher than nonbullies. Among the bully group, males scored higher than females on all subscales of Conners’ Teacher Rating Scale-28. Among the nonbully group, males scored higher than females on all subscales except passive inattentiveness.

**Table 4:** The Conners’ Teacher Rating Scale-28 categories among the studied groups regarding gender

	Total participants (*N* = 70)	Bully group (*N* = 35)			Non-bully group (*N* = 35)			*P* value for males	Chi-square for males	*P* value for females	Chi-square for females	*P* value for both groups	Chi-square for both groups
		Males	Females	Total of studied group	Males	Females	Total of control group						
Conduct problem	55 (78.6%)	23 (100%)	11 (91.7%)	34 (97.1%)	13 (56.5%)	8 (66.7%)	21 (60%)	0.001*	12.7	0.3	2.2	0.000*	14.33
Passive-inattentive	31 (44.3%)	14 (60.9%)	7 (58.3%)	21 (60%)	5 (21.7%)	5 (41.7%)	10 (28.5%)	0.01*	7.26	0.6	0.66	0.008*	7.006
Hyperactivity index	37 (52.9%)	18 (78.3%)	8 (66.7%)	26 (74.2%)	7 (30.4%)	4 (33.3%)	11 (31.4%)	0.003*	10.6	0.2	2.66	0.000*	12.8

*significant *p* value

We assessed the correlation between Conners’ Teacher Rating Scale-28 scores with age, grade, and bullying types. There was a moderate positive correlation between conduct disorder with age and grade (Table 5).

**Table 5:** Correlation of Conners’ Teacher Rating Scale-28 with age, grade and bullying types

	Age	Grade	Verbal	Psychological	Social	Physical
Conduct disorder						
*r*	0.678	0.678	0.056	− 0.046	− 0.043	− 0.242
*P* value	0.000*	0.000*	0.751	0.793	0.807	0.161
Passive inattentive passive inattentive						
*r*	0.015	0.015	0.119	0.029	− 0.090	0.001
*P* value	0.932	0.932	0.494	0.867	0.606	0.996
Hyperactivity index						
*r*	0.063	0.063	0.218	0.050	− 0.106	0.230
*P* value	0.717	0.717	0.209	0.774	0.545	0.184

*significant *p* value

## Discussion

Bullying involves the repetitive and deliberate use of power by a person or group against another, leading to physical or psychological harm [29]. In this cross-sectional study, we assessed the prevalence rate and risk factors for school bullying among 280 primary school students (10–12 years old). Additionally, we examined the relationship between bullying with ADHD and conduct disorder.

A slightly higher proportion of males (52.1%) than females (47.9%) participated in our study. Numerous studies of bullying behaviors recruited male students more than females [30–32], but an equal number of boys and girls was recruited by [33]. Several studies investigated bullying among children 11–15 years old [31, 33–35]. Other studies were conducted among higher age groups of 12–18 years [30, 32, 36]*.*

Significant bullying was observed in 12.5% of participants in the present study. A previous Egyptian study on 476 students in Giza governorate reported a 9.5% prevalence of school bullying [30]. These differences might be explained by the different mean ages between studies (14.4 ± 1.8 vs. 11.08 ± 0.8 years in the current study) and different measures and tools used in the studies. National surveys conducted in 40 Western countries reported rates of involvement in bullying ranging from 4.8 to 45.2% [34]*.* A cross-sectional study on 1192 middle school students in Nigeria reported a 59.9% prevalence rate of bullying behavior [36]. Different methods and cultural differences in defining the problem, as well as differences in the target populations and instrumentation used, could account for the wide range of prevalence rates found across countries.

Regarding forms of bullying, this study found that verbal and social bullying were most prevalent among our study group, followed by psychological and physical bullying. This finding is consistent with [32], who reported that verbal bullying was most common, followed by social and then physical bullying among US adolescents from grade 6 to grade 10*.* Similarly, a study on forms of bullying among secondary school students aged 13 and 15 in Finland found that the most common forms of bullying were verbal teasing and social exclusion [31]*.*

The current study found statistically significant differences between males and females in terms of bullying types. Males scored higher in verbal, psychological, and physical bullying, but females scored higher in social bullying. This finding can also be explained by cultural factors, as boys in Egyptian communities, especially Upper Egypt, are less often punished for misbehavior compared to girls. A similar finding was reported in a study of bullying behaviors among 8- to 15-year-olds of children in Finland. For direct aggression, men outperformed women, while for indirect aggression, women outperformed men [33]*.* Another study among US adolescents found that males were more involved in physical and verbal bullying, whereas females were more involved in social bullying [32]. Ref. [37] reported that males scored higher on the bullying scale among a sample of 454 public school students in Mississippi, USA. Another Egyptian study that assessed the prevalence and risk factors of violence among elementary school children in Cairo found that all forms of violence were higher among males [35]. When it comes to bullying, boys are more likely than girls to engage in overt forms of aggression (like physical bullying), while girls are more likely to engage in subtle forms of aggression (like gossiping, teasing, rejecting, verbal threatening, and humiliating) that can be difficult to identify [38]. The difference between these results and those of the present study might be explained by the different study settings as well as different tools used for assessment.

In the current study, we examined individual, peer, and school risk factors for bullying behavior. The most significant risk factors for bullying were attention-seeking and imitating bad behavior of others, followed by not being accepted by peers, having a negative attitude toward teachers, and joining bad peer groups. Compared with the nonbully group, many risk factors were significantly more prevalent among students in the bully group.

Most individual risk factors were significantly more prevalent among the bully group, except low scholastic achievement and underweight body build. A previous study reported that students involved in bullying and victimization are less academically engaged [39]. Additionally, studies have found a strong link between previous academic failure and bullying, which could be explained by the fact that student suppression as a result of academic failure has a negative impact on their behavior [30]*.*

The prevalence of peer risk factors (except being bullied) was significantly higher in the bully group than the nonbully group. According to Salmivalli and his colleagues [40], students who were part of a bullying peer group had higher rates of bullying themselves. In contrast, aggressive youths are less likely to be influenced by their friends because they have already established a habit of aggression, according to Larsen and his colleagues [41]. Moreover, a multivariate, multilevel analysis of middle school students in New Brunswick, Canada, clearly indicated that the relationship between bully and victim was reciprocal [42].

The prevalence of school risk factors was also significantly higher among the bully group than the nonbully group. Bullying victims may express their frustrations on teachers or other students if they are punished frequently at school, which can encourage aggressive behavior [43]. Students who are subjected to physical punishment are more likely to engage in violent behavior [35]. Moreover, students who were exposed to punishment at school reported higher rates of being bullied [30].

In the present study, the bully group scored significantly higher than the nonbully group on all subscales of Conners’ Teacher Rating Scale-28. Additionally, male bullies scored significantly higher on all subscales compared to nonbully males. Similar results were observed among female groups (bully and nonbully groups); female bullies scored significantly higher on all subscales except conduct problems. Our findings are in line with those of a study that assessed the association between ADHD with bullying in a population of 577 fourth graders in Sweden, which found that children diagnosed with ADHD were involved in bullying more often than other children [21]. Children with ADHD are at elevated risk of being involved in bullying either as aggressors or as victims in comparison with typical children, and ADHD is considered a risk factor for bullying [20, 22, 44]*.* Furthermore, a previous study conducted to evaluate the relationship between bullying and psychiatric disorders among 420 children in Finland found that ADHD, oppositional/conduct disorder, and depression were the most common psychiatric disorders among children involved in bullying [19]. Zablotsky and his colleagues [45] also found that children identified as bully-victims were more likely to have ADHD, conduct disorder, or oppositional defiant disorder than typical children, and the frequency of bullying behaviors was significantly associated with the level of psychological impairment.

Contrary to what was observed in males, the present study revealed that no statistically significant association was found between bullying and conduct, hyperactivity, passive inattentiveness, or hyperactivity index in females. This finding can be explained by the small number of affected females, which made it difficult to demonstrate the difference between groups. Moreover, girls with ADHD appear to be less severely affected than boys, with fewer comorbid externalizing and internalizing behaviors [46]*.*

This study has a few limitations, such as the relatively small sample size especially female’s gender. Additionally, the current study investigated only traditional bullying, and the sample was selected from students in the fourth, fifth, and sixth grades in one primary school. Effects of grade level, socioeconomic status, family, and community risk factors for bullying were not included. This study relied on participants’ honesty and accurate self-reporting to screen for bullying. Some students might have been embarrassed to admit involvement in bullying, which could have impacted the results. The 2019 coronavirus disease pandemic resulted in school closures, which limited the continuation of this study to obtain more data, such as IQ testing for the students.

It is recommended that a bullying prevention committee be formed at school that comprised all school personnel, to address various factors associated with bullying behavior. Additionally, ADHD diagnosis and treatment strategies should be incorporated as effective interventions for bullying. Future researchers are encouraged to replicate this study on a larger scale, addressing factors that were not addressed in this study, such as other individual, family, and community risk factors. Future studies can compare gender differences in both traditional and cyber-bullying among samples of students in more grade levels and more schools.

## Conclusions

It can be concluded from this study that male students in primary schools show higher degrees of bullying with a significant association between bullying, conduct, and ADHD. This study also demonstrated a relationship between ADHD and bullying at school.

## Data Availability

All data generated or analyzed during this study are available from corresponding author on request.

## Abbreviations

ADHD: Attention deficit hyperactive disorder
K-SADS: The Kiddie Schedule for Affective Disorders and Schizophrenia
